# Analysis of Late-Onset Ovarian Insufficiency after Ovarian Surgery: Retrospective Study with 75 Patients of Post-Surgical Ovarian Insufficiency

**DOI:** 10.1371/journal.pone.0098174

**Published:** 2014-05-23

**Authors:** Seido Takae, Kazuhiro Kawamura, Yorino Sato, Chie Nishijima, Nobuhito Yoshioka, Yodo Sugishita, Yuki Horage, Mamoru Tanaka, Bunpei Ishizuka, Nao Suzuki

**Affiliations:** 1 Department of Obstetrics and Gynecology, St. Marianna University School of Medicine, Kawasaki city, Kanagawa prefecture, Japan; 2 Department of Advanced Reproductive Medicine, St. Marianna University School of Medicine, Kawasaki city, Kanagawa prefecture, Japan; The Chinese University of Hong Kong, Hong Kong

## Abstract

The primary objectives of the present study are to determine the period of onset of ovarian insufficiency after surgery and to confirm potential risk factors for ovarian insufficiency after surgery for the removal of benign ovarian cysts. Data were obtained from 75 patients who underwent surgery for benign ovarian cysts prior to the onset of ovarian insufficiency. Our analysis included 835 ovarian insufficiency patients who were referred to our institution from July 2003 to July 2013. Several epidemiological parameters of ovarian insufficiency after surgery (age at operation, period of onset of ovarian insufficiency, operation procedure, and pathological diagnosis) were investigated. Of the 835 patients who had ovarian insufficiency, 75 patients (9.0%) underwent ovarian surgery before the onset of ovarian insufficiency. Of those 75 patients, 66 patients (88.0%) underwent cystectomy. For the majority of the 75 patients the surgical indication was the presence of endometriotic cysts (57 patients; 76.0%). Twelve patients (16.0%) underwent multiple surgeries (all bilateral cystectomies). The mean age of the patients at the time of surgery was 27.8±5.5 years-old, and the mean period of onset of ovarian insufficiency was 5.8±3.8 years. In patients with cystectomy, the patient's age at the time of surgery and period of onset of ovarian insufficiency was well-correlated (coefficient of correlation; hemilateral endometriotic cystectomy: −0.64, bilateral endometriotic cystectomy: −0.61, and multiple endimetriotic cystectomy: −0.40). We found that cystectomy of endometriotic cysts is the potential risk factor for ovarian insufficiency after surgery, at times, the onset of ovarian insufficiency long after cystectomy. Therefore, it is important to monitor ovarian reserve for an extended period of time after ovarian surgery. It is particularly important to monitor ovarian reserve long-term for patients who wish to conceive in the future and to suggest a variety of infertility treatments appropriate for their ovarian reserve.

## Introduction

Recently, the advancement of surgical techniques has been remarkable, especially the development of laparoscopic surgical techniques for gynecological diseases and laparoscopy is now considered to be the gold standard for the treatment of benign ovarian cysts. Due to the development of laparoscopic surgical techniques, the _ENREF_1invasiveness of ovarian surgery is greatly decreased; however, some concerns remain over diminished ovarian reserve post-cystectomy. The deteriorate effects of cystectomy on ovarian function has recently been discussed [1]–[6]. In particular, researchers questioned the use of the stripping technique during cystectomy because it results in the removal of normal ovarian tissue along with the wall of endometriotic cysts, resulting in the loss of the follicular pool [7]–[9]. The incidence of ovarian insufficiency after laparoscopic excision of bilateral endometriotic cysts is estimated to be around 1.3–2.4% [4], [6], whereas contralateral ovarian cystectomy did not induce severe ovarian damage [4]. In addition, after ovarian cystectomy, the serum anti-Müllerian hormone (AMH) levels in patients with endometrioma were lower than those in nonendometrioma [3]. Although ovarian insufficiency was reported to occur immediately after cystectomy [6], ovarian dysfunction may become apparent after a certain lag period following surgery.

Premature ovarian failure (POF) is classically defined as 4–6 months of amenorrhea in women under 40 years of age who also exhibit elevated serum follicle-stimulating hormone (FSH) and low estradiol hormone levels [10]. However, recent studies have revealed two types of ovarian insufficiency–patients with amenorrhea or irregular menstrual cycle are designated as “overt type” while patients with a regular menstrual cycle are designated as “biochemical type” [10].

In this study, we sought to investigate the period of onset of ovarian insufficiency after surgery. Ovarian insufficiency was defined as continuously elevated gonadotropin and low AMH levels before 40 years of age, whether menstruating or in amenorrheic. Furthermore, we sought to confirm the potential risk factors for ovarian insufficiency after surgery by evaluating both the type of ovarian cysts and the surgical methods employed.

## Materials and Methods

### Patients

Data were retrospectively obtained from the clinical records of patients who were referred to the Primary Ovarian Insufficiency Unit at the Center for Reproductive Medicine, Department of Obstetrics and Gynecology of the St. Marianna University School of Medicine Hospital from July 2003 to July 2013. This study was approved by the institutional review board of Saint Marianna University (study registration number: 571), and we received written informed consents from all participants to having their data used this research in keeping with the Declaration of Helsinki. Study participants were Japanese females under the age of 40, who underwent surgery for ovarian cysts prior to the onset of ovarian insufficiency and had regular menstrual cycles before undergoing surgery. The patients were categorized into four groups on the basis of surgical treatment: oophorectomy (patients with hemi-lateral oophorectomy), hemilateral cystectomy, bilateral cystectomy (patients who underwent bilateral cystectomy in a single operation), and multiple surgeries (patients who underwent repeated surgeries resulting in bilateral cystectomy). Additionally, these patients were classified on the basis of their pathological diagnosis: endometriotic cysts (EMC) or non-endometriotic cysts (other).

Patients with the following conditions were excluded from the study due to the innate potential of these conditions to induce ovarian insufficiency apart from any surgical intervention [11]: autoimmune disease, chromosomal abnormality, endocrine diseases, gonadotoxic treatment by systemic chemo- or radiation therapy prior to surgery. Patients who underwent surgery prior to menarche were also excluded because it was not possible to evaluate normal ovarian function before surgery in these patients.

### Data Collection

Drawing from clinical records, data were collected on the following epidemiological parameters for ovarian insufficiency: the patient's age at operation, time elapsed from surgery to the onset of ovarian insufficiency, operation procedure, serum AMH levels at the patient's first visit to the hospital, and pathological diagnosis of ovarian cysts. Serum AMH levels were measured using a commercial assay kit (AMH Gen II ELISA, Beckman Coulter, Brea, CA) according to the manufacture's protocol.

### Statistical Analysis

Data analysis was performed using the Statistics Package for Social Sciences (SPSS 11.0, Chicago, IL). Qualitative data were expressed as numbers and compared using the chi-squared test or Kruskal-Wallis test. To investigate correlation between age at operation and the period of onset of ovarian insufficiency, we used a non-parametric Spearman's correlation coefficient by rank test; *P*<0.05 was considered statistically significant.

## Results

### Epidemiological characteristics of ovarian insufficiency after surgery

Among 835 patients with ovarian insufficiency, 75 patients (9.0%) underwent ovarian surgery before the onset of ovarian insufficiency. The characteristics of ovarian insufficiency are shown in Table 1. Of those 75 patients, 66 patients (88.0%) underwent an ovarian cystectomy, whereas only 9 (12.0%) patients underwent hemilateral oophorectomy. Of note, no patient underwent a bilateral oophorectomy. The pathological analyses revealed that 57 patients (76.0%) had endometriotic cysts, whereas 18 patients (24.0%) had other types of benign cysts (e.g. dermoid cyst or serous or mucinous cystadenoma). Of the 57 patients with endometriotic cysts, 11 patients underwent multiple surgeries with repeated cystectomy, 27 patients underwent bilateral cystectomy, 16 patients underwent a hemilateral cystectomy, and 3 patients underwent hemilateral oophorectomy. Based on a pelvic examination during laparoscopic surgery for oophorectomy, these patients were classified as stages III-IV following the revised American Society for Reproductive Medicine (rASRM) classification of endometriosis. Of the patients with non-endometriotic cysts (18 patients), 5 patients underwent bilateral cystectomy, 6 patients underwent hemilateral cystectomy, and 6 patients underwent hemilateral oophprectomy. Of these, only one patient underwent multiple surgeries. Among the patients with endometriotic cysts, the occurrence of ovarian insufficiency after surgery was not significantly different between patients who were treated with the different surgical methods (laparotomy versus laparoscopic surgery) (Table 2). Although we analyzed serum AMH levels in 52 of the 75 patients, the levels were below the limit of detection of our assay (<0.16 ng/ml) and all patients exhibited continuously elevated FSH levels (>40 IU/ml).At more than 4 months after surgery, amenorrhea was present in 72 patients (96.0%).

**Table 1:** Characteristics of ovarian insufficiency after ovarian surgery.

	Oophorectomy		Cystectomy					
	Hemilateral		Hemilateral		Bilateral		Multiple Surgery (All bilateral cystectomy)	
Type	EMC	Other	EMC	Other	EMC	Other	EMC	Other
Total number	9		66					
Number	3	6	16	6	27	5	11	1
Age at the time of surgery (years old)	28 (±6.2)	22 (±4.1)	27.2 (±5.5)	28.9 (±7.1)	28.8 (±5.3)	23 (±3.3)	30.7 (±3.6)	26
Period of onset (years)	7 (±6)	6.3 (±2.6)	6.3 (±4.3)	4.5 (±4.6)	6.3 (±3.7)	7.8 (±3.4)	3.6 (±2.5)	33
Procedure L	3	6	8	5	14	3	5	1
Procedure LS	0	0	8	1	13	2	6	0

Values are expressed mean ±SD.

EMC = endometriotic cysts, other = non-endometriotic cysts.

L = laparotomy, LS = laparoscopic surgery.

**Table 2:** Characteristics of ovarian insufficiency after endometriotic cystectomy.

	Endometriotic Cystectomy			*P* value
	Hemilateral	Bilateral	Multiple (Bil)	
Number	16	27	11	
Age at the time of surgery (years old)	27.1 (±5.5)	28.9 (±5.3)	30.7 (±3.6)	*P* = 0.14
Period of onset (years)	6.3 (±4.3)	6.3 (±3.7)	3.6 (±2.5)	*P* = 0.11
Procedure L	8	14	5	*P* = 0.93
Procedure LS	8	13	6	

Values are expressed mean ±SD.

EMC = endometriotic cyst Bil = Bilateral endometriotic cyst.

L = laparotomy, LS = laparoscopic surgery.

Qualitative data were expressed as numbers and compared using the chi-squared test or Kruskal-Wallis test. (*P*<0.05).

### The period of onset of ovarian insufficiency after surgery

**Figure 1:**
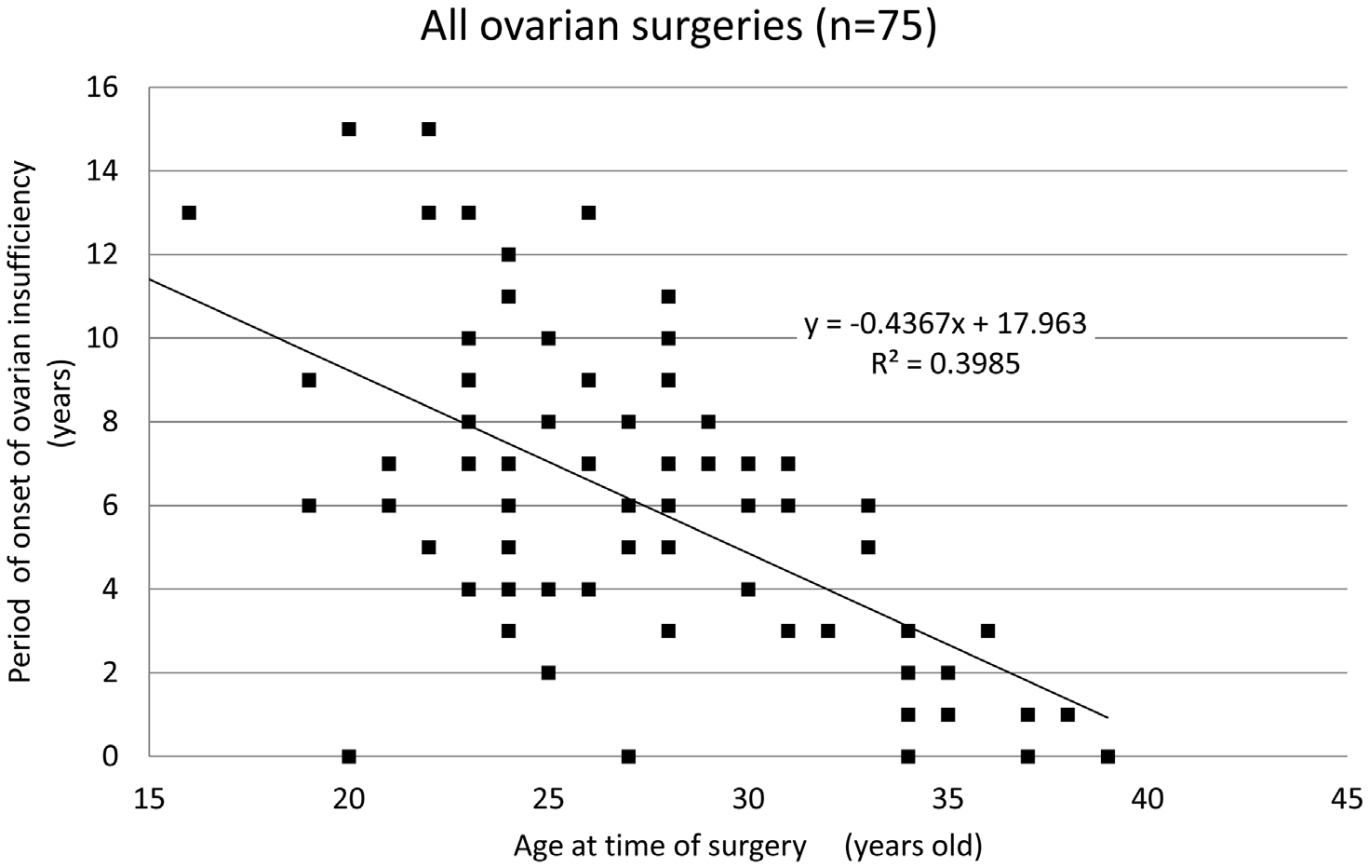
The period of onset of ovarian insufficiency post-ovarian surgery for each patient by age. A strong correlation was observed between age at the time of surgery and the period of onset of ovarian insufficiency (correlation coefficient:−0.63, Spearman‘s correlation coefficient by rank test).

**Figure 2:**
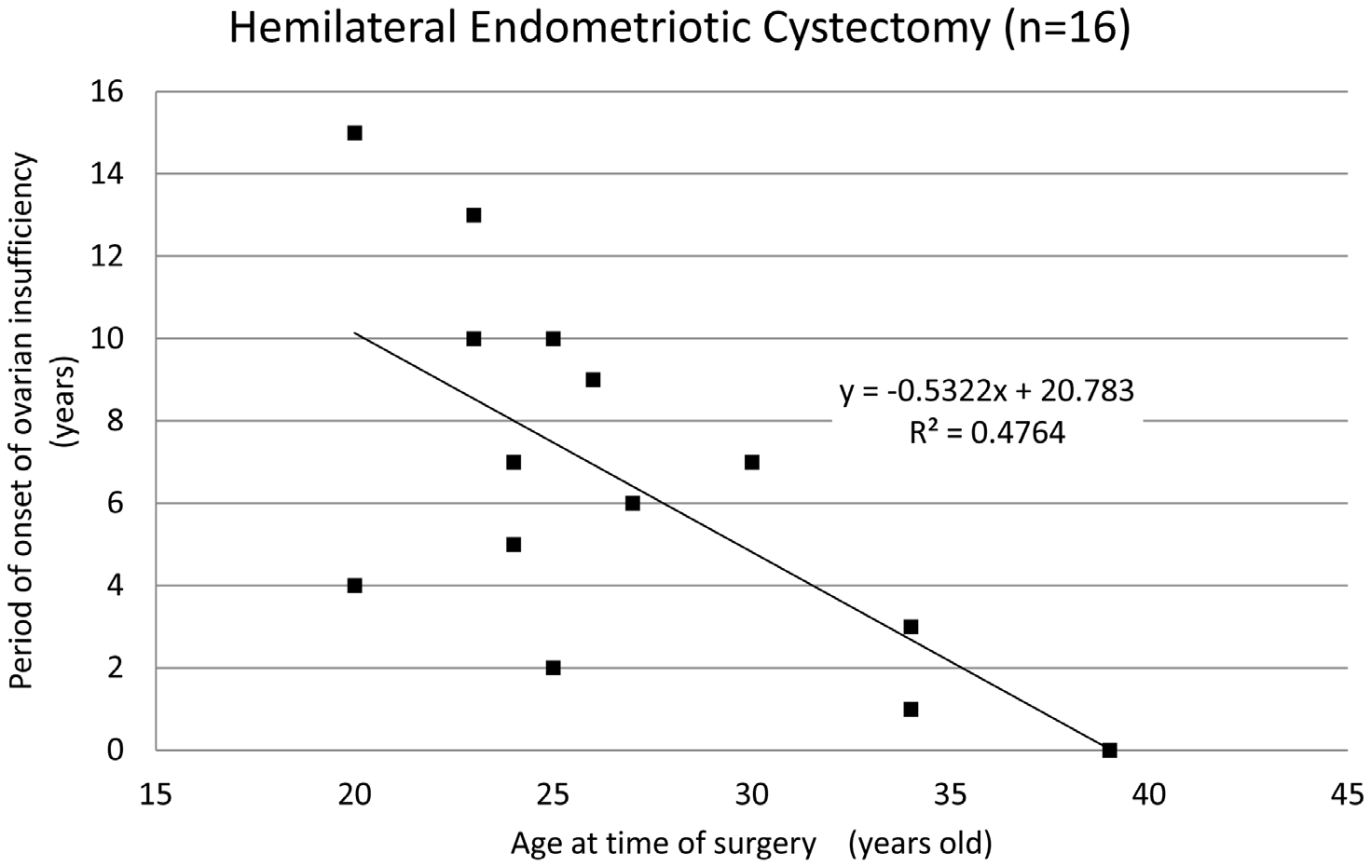
The period of onset of ovarian insufficiency after hemilateral endometriotic cystectomy. A strong correlation was observed between age at the time of cystectomy and the period of onset of ovarian insufficiency (correlation coefficient:−0.64, Spearman‘s correlation coefficient by rank test).

**Figure 3:**
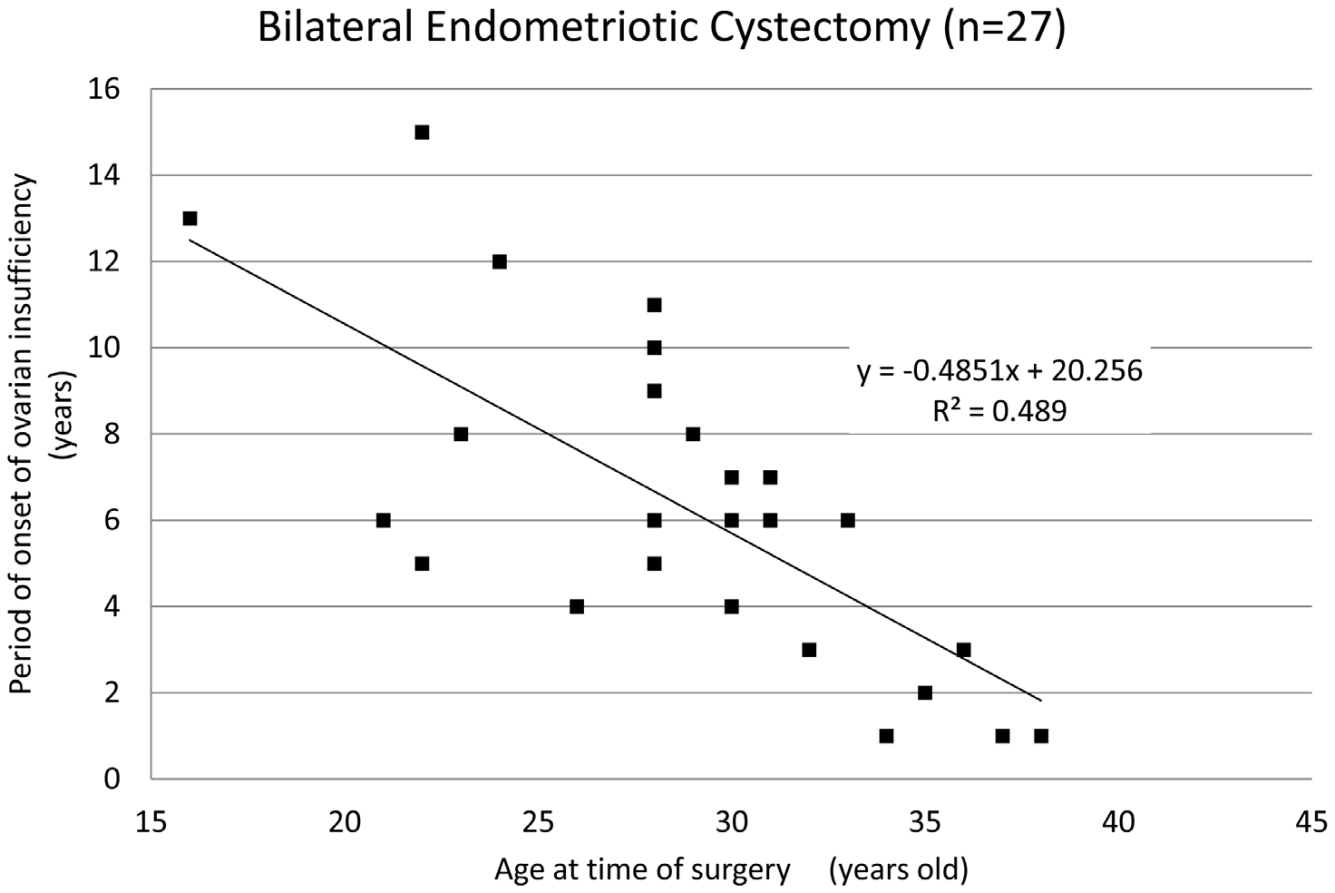
The periof of onset of ovarian insufficiency after bilateral endometriotic cystectomy. A strong correlation was observed between age at the time of cystectomy and period of onset of ovarian insufficiency (correlation coefficient:−0.61, Spearman‘s correlation coefficient by rank test).

**Figure 4:**
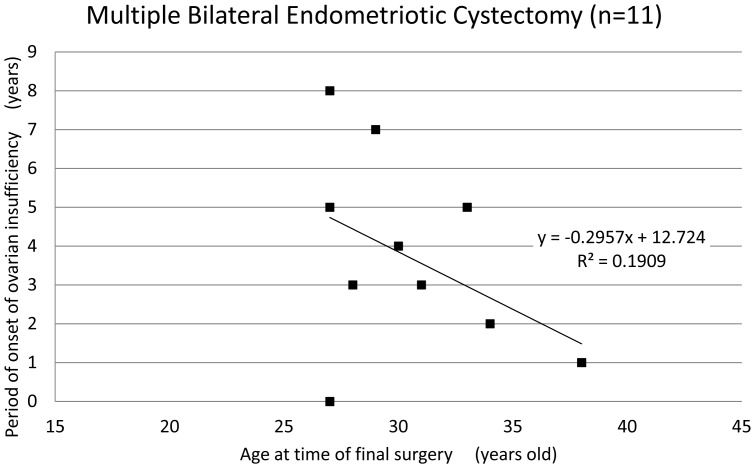
The period of onset of ovarian insufficiency after the last multiple endometriotic cystectomy. A weak correlation was observed between age at the time of the patient's last surgery and the period of onset of ovarian insufficiency (correlation coefficient:−0.40, Spearman‘s correlation coefficient by rank test).

The mean age of patients at the time of ovarian surgery was 27.8±5.5 years and the mean period of onset of ovarian insufficiency was 5.8±3.8 years after surgery. The period of onset of ovarian insufficiency was defined as the time period from surgical intervention until amenorrhea or a diagnosis of ovarian insufficiency occurred. Interestingly, there was a correlation between the age of patients at the time of surgery and the period of onset of ovarian insufficiency (Fig. 1, coefficient of correlation: −0.63). Among the 66 patients who underwent cystectomy, only 4 patients (6.1%) displayed ovarian insufficiency within one year of surgical treatment, and most patients (93.9%) only exhibited ovarian insufficiency after a certain lag period post-cystectomy. Though no significant difference was found in the mean age and the period of onset of ovarian insufficiency (Table 2), there was a strong correlation between the age of the patient at the time of cystectomy and period of onset of ovarian insufficiency (Figs. 2 and 3, coefficient of correlation; hemilateral endometriotic cystectomy: −0.64 and bilateral endometriotic cystectomy: −0.61). In patients who underwent multiple endometriotic cystectomies there was a weak correlation between the patient's age at the time of their final operation and the period of onset of ovarian insufficiency (Fig. 4, coefficient of correlation: −0.40).

## Discussion

In present study, the mean period of onset of ovarian insufficiency after cystectomy was approximately 6 years, signaling the importance of long-term follow-up after surgery to detect ovarian insufficiency. This is especially true for young patients due to the long lag period between surgery and the onset of ovarian insufficiency. Because the onset of ovarian insufficiency was more than 10 years after surgical intervention in some young patients, a lengthier follow-up is needed in younger patients. It is important to note that the majority of patients are administered hormone treatments to delay hormone withdrawal bleeding, thus making it difficult to determine the onset of amenorrhea. Therefore, to detect ovarian insufficiency, we used the following criteria: continuously elevated gonadotropin and below-detection levels of AMH (<0.16 ng/ml) in those under 40 years of age, whether menstruating or amenorrheic.

Enrolled patients were not evaluated for ovarian reserve before surgical intervention, as determined by serum AMH levels, but these patients had regular menstrual cycles. The period of onset of ovarian insufficiency after surgery was long, suggesting the involvement of a few patients with impaired ovarian reserve in present study. An earlier report by Busacca et al. found that ovarian insufficiency appeared immediately after cystectomy during a follow-up period of 4.6±2.7 years, based on a retrospective study of 126 patients (mean age: 30.4±4.3 years-old) with regular menstrual cycles (102 patients) who underwent bilateral endometriotic cystectomy [6]. In an earlier study by another group, post-surgical ovarian failure was defined by the absence of menses for one or more years with elevated gonadotropin levels [6]. However, it is difficult to directly compare these results from the Busacca group to our study due to the differences in study design. For example, the mean period of onset of ovarian insufficiency after cystectomy shown in the Busacca et al. study might have been longer if they had observed patients for more than 6 years. Of note, some patients exhibited ovarian insufficiency within one year in our study, thus emphasizing the importance of initiating extensive studies on ovarian function shortly after surgery. In patients with multiple surgeries, the mean period of onset of ovarian insufficiency tended to be shorter than with other groups and in these patients there was no correlation between patient's age and the mean period of onset of ovarian insufficiency. Thus, repeated ovarian surgeries could exacerbate the adverse effects of surgery on ovarian function compared with other parameters.

It has been demonstrated that ovarian surgery may cause ovarian insufficiency [11],and it is well known that women with endometriotic cysts have an increased risk of ovarian insufficiency, particularly if bilateral cysts have been removed [3], [4], [6], [10]. Consistent with earlier reports, the proportion of patients with endometriotic cysts in the present study was high compared with that of non-endometriotic cyst patients. The presence of endometriosis has been shown to be associated with a decrease in the follicular ovarian reserve accompanied by a reduction in AMH levels [3], [12], [13]. These findings coincide with our results demonstrating that endometriotic cysts are a major pathological origin for ovarian insufficiency after surgery. On the contrary, non-endometriotic cysts have an anatomical capsule allowing for ease of enucleation during surgery; therefore, the loss of functional follicular reserve is minimal after cystectomy [14]. Nonetheless, we discovered some patients had ovarian insufficiency in cases of non-endometriotic cysts, suggesting that other surgical interventions might also affect ovarian function.

It became apparent that patients could develop ovarian insufficiency long after surgical intervention. Consistent with previous studies [4], [6], cystectomy of endometriotic cysts is the leading cause for ovarian insufficiency in our cases, although any surgical procedure on the ovary may induce ovarian insufficiency. Thus, surgeons should be aware of the risks of ovarian surgery for future ovarian dysfunction and communicate this information to their patients. Also, it is important to conduct long-term follow-up to detect ovarian dysfunction at an early stage before it leads to untreatable complete ovarian insufficiency.
